# Supplementary material for: Design thinking teaching and learning in higher education: Experiences across four universities
Source: PLoS One. 2022 Mar 24;17(3):e0265902. doi: 10.1371/journal.pone.0265902 (PMC8947127; doi:10.1371/journal.pone.0265902)
Supplement: S1 Table — (DOCX) [file pone.0265902.s001.docx]

**S1 Table. Outcomes of Design Thinking Coursework**

| **Outcomes of Design Thinking Coursework**  *Please note how often, as a direct result of this specific course, you observed the following outcomes:* | **Faculty** (n=19)  Mean (SD) | **Students**  (n=179)  Mean (SD) | **Lake, et al. [21]**  (n=35)  Mean (SD) | **Liedtka & Bahr [24]**  (n=416)  Data not available |
| --- | --- | --- | --- | --- |
| 1. Created a deeper understanding of stakeholder needs | 4.33 (.59) | 4.04 (.81) | 3.57 (---) | --- |
| 1. Helped me see the problems in new ways, resulting in solving more promising problems | 3.89 (.83) | 4.31 (.73) | -- | X |
| 1. Helped me find alignment across different perspectives | 3.67 (.77) | 4.18 (.75) | ---- | --- |
| 1. Enhanced my ability to pivot when initial solutions didn't work | 3.89 (.67) | 4.09 (.86 | --- | X |
| 1. Increased my engagement in the class | 3.94 (.54) | 4.09 (1.00) | --- | --- |
| 1. Leveraged diversity on team to find more creative solutions | 3.28 (.58) | 3.98 (.89) | 2.89 (---) | --- |
| 1. Built new relationships locally that continued after the initial project was completed | 2.83 (.86) | 3.35 (1.24) | --- | X |
| 1. Expanded access to new resources for individuals and teams | 3.50 (1.04) | 3.72 (1.02) | 2.89 (---) | X |
| 1. Helped pool resources for greater impact | 3.44 (1.04) | 3.87 (.98) | 3.29 (---) | X |
| 1. Helped to build alignment across diverse stakeholders | 3.33 (1.09) | 3.56 (1.06) | --- | --- |
| 1. Enhanced other stakeholders willingness to collaborate on new solutions | 3.06 (1.06) | 3.50 (1.14) | --- | X |
| 1. Helped to surface critical assumptions being made about new ideas so that they could be tested | 3.44 (.86) | 3.83 (.91) | 3.21 (---) | --- |
| 1. Helped teams to gather more accurate feedback on ideas from users and other stakeholders | 3.50 (1.04) | 3.99 (.86) | --- | --- |
| 1. Built trust among team members | 3.56 (.89) | 4.22 (.04) | 3.29 (---) | X |
| 1. Built trust between problem-solving teams and other stakeholders | 3.33 (.97) | 3.85 (1.03) | 2.47 (---) | X |
| 1. Improved the ability to talk to each other in ways that produced better outcomes | 4.00 (.59) | 4.23 (.75) | 3.21 (---) | --- |
| 1. Allowed new and better solutions, not visible at the beginning of the process, to emerge during it | 4.00 (.69) | 4.35 (.72) | 3.34 (---) | X |
| 1. Fostered the inclusion of user input | 4.06 (.64) | 4.05 (.99) | --- | X |
| 1. Reduced the risk of pursuing ideas that will likely fail | 3.50 (.62) | 3.81 (.90) | 2.71 (---) | --- |
| 1. Helped people involved to examine their own biases and preconceptions | 3.72 (.90) | 3.83 (.97), | 3.32 (---) | X |
| 1. Helped champions for new ideas who were enthusiastic about their implementation emerge during the Design Thinking process | 3.22 (.88) | 3.93 (.98) | --- | --- |
| 1. Increased people's willingness to take action | 3.83 (.51) | 3.95 (.88) | --- | --- |
| 1. Created a sense of safety to try new things | 3.78 (.65) | 4.10 (.90) | 3.06 (---) | X |
| 1. Allowed for involvement of key stakeholders who were not on the core team | 3.22 (1.35) | 3.63 (1.10) | --- | --- |
| 1. Helped teams to persist despite challenges along the way | 3.89 (.68) | 4.04 (.90) | 3.09 (---) | --- |
| 1. Gave people more confidence in their own creative abilities | 3.94 (.64) | 4.20(.78) | --- | X |
| 1. Improved the creativity of new solutions | 4.11 (.47) | 4.26 (.74) | --- | --- |
| 1. Improved the likelihood of the implementation of new solutions | 3.56 (1.04) | 4.03 (.85) | 3.00 (---) | X |
| 1. Made working together more enjoyable | 4.17 (.62) | 4.17 (.97) | 3.40 (---) | --- |
| 1. Made it easier to discard solutions that didn't work as planned | 3.56 (.51) | 3.99 (.82) | 2.97 (---) | X |
| 1. Helped people interested in trying new things to connect and support each other | 3.56 (.62) | 3.97 (.89) | --- | X |
| 1. Encouraged people's open-mindedness to try new things | 3.83 (.51) | 4.30 (.76 | --- | X |
| 1. Encouraged shifts in organizational culture that made it more customer-focused | 3.28 (1.02) | 3.94 (1.02) | --- | X |
| 1. Encouraged changes in organizational culture that made risk-taking more acceptable | 3.72 (.96) | 3.99 (.97) | --- | X |
| 1. Equipped team members with new capabilities that can be applied to other projects | 4.11 (1.02) | 4.28 (.84) | 3.69 (---) | --- |
| 1. Kept people motivated to work on a project to achieve impact | 3.94 (1.00) | 4.06 (.84) | --- | X |
| 1. Broadened organization's definition of what innovation is | 3.33 (.97) | 3.97 (.91) | --- | X |
| 1. Created a common language/framework among team members | 4.11 (.67) | 4.18 (.86) | 3.40 (---) | --- |
| 1. Increased willingness to collaborate with others (cross-functionally and cross-departmentally) | 3.56 (.92) | 4.14 (.91) | --- | --- |
| 1. Increase a sense of ownership and acceptance of a solution | 3.78 (.55) | 4.14 (.83) | --- | X |
| 1. Increased appreciation for use of data to help drive decisions | 3.50 (.62) | 4.10 (.87) | --- | X |
| 1. Increased engagement of teammates involved in the design thinking process | 3.94 (.54) | 4.21 (.82) | --- | X |
| *Cronbach Alpha* | .96 | .96 | --- | --- |

***SD*** *= Standard Deviation; All items measured on a scale from 1-Never to 5-Almost Always*

*“—" indicates item was not included on survey and/or value was not reported.*

*“X” indicates that item was included on survey but descriptive statistics were not reported.*
